# Pancytopenia and Acute Liver Failure Caused by Mild COVID-19 in an Older Patient: A Case Report

**DOI:** 10.7759/cureus.72681

**Published:** 2024-10-30

**Authors:** Issei Tanaka, Yutaka Sato, Chiaki Sano, Ryuichi Ohta

**Affiliations:** 1 Family Medicine, International University of Health and Welfare Graduate School of Health Sciences, Tokyo, JPN; 2 Community Care, Unnan City Hospital, Unnan, JPN; 3 Community Medicine Management, Shimane University Faculty of Medicine, Izumo, JPN

**Keywords:** acute liver failure, covid-19, family medicine, general medicine, multisystem inflammatory syndrome, myelosuppression, pancytopenia, rural, steroids

## Abstract

An 89-year-old woman, living independently, presented to a community hospital with complaints of back pain and anterior chest pain after a fall at home. During her hospitalization, she was infected with COVID-19, and although her fever temporarily resolved with symptomatic treatment, she developed pancytopenia and liver dysfunction along with fever again. Blood tests and imaging studies ruled out acute cholangitis or hepatitis virus infection, and a diagnosis of myelosuppression and liver dysfunction due to multi-system inflammatory syndrome (MIS) was made. Treatment with prednisolone was initiated, and improvement in liver enzyme levels and pancytopenia was observed, indicating that steroid therapy was effective. Still, after steroid discontinuation, the patient again presented with pancytopenia. This case demonstrates the potential of multiorgan damage that can occur after a COVID-19 infection and emphasizes the importance of tapering and monitoring steroid therapy, especially in the management of myelosuppression and liver dysfunction due to MIS, during a COVID-19 infection. This may contribute to improved patient prognosis.

## Introduction

COVID-19 has been associated with a broad spectrum of systemic complications, affecting multiple organs and systems within the body [1]. While complications involving the lungs, heart, and brain have been extensively studied and widely recognized, COVID-19 can also directly impact the liver and hematologic systems, leading to conditions such as acute liver failure and pancytopenia, albeit less commonly [2,3]. Liver injury in COVID-19 patients can result from direct viral infection of hepatocytes, immune-mediated injury, or drug-induced hepatotoxicity during treatment. Additionally, COVID-19 may induce pancytopenia due to mechanisms like bone marrow suppression, immune-mediated cytopenias, or cytokine storm [4].

These conditions can emerge at varying stages of the disease, with liver failure sometimes manifesting even after the acute phase has resolved. This delayed impact on multiple organs is part of a broader phenomenon known as multisystem inflammatory syndrome (MIS), where organs, including the liver, may be affected several weeks post-infection [5].

In this case report, we present the case of an 89-year-old patient who initially experienced mild COVID-19 symptoms, followed by the development of acute liver failure and pancytopenia. The patient's condition responded positively to treatment with prednisolone, highlighting a possible therapeutic approach for these rare but severe complications. This report provides a detailed overview of the management strategies, addressing the challenges and considerations in treating COVID-19-related acute liver failure and pancytopenia.

## Case presentation

An 89-year-old woman living independently presented to a community hospital with a chief complaint of chest pain. Three days before her visit, she experienced back pain and anterior chest pain following a fall at home and was brought to the emergency room. Her medical history included hypertension, dyslipidemia, and iron deficiency anemia. She was on medications, including Olmesartan 20 mg, amlodipine 5 mg, atorvastatin 5 mg, and ferrous sodium citrate 50 mg daily.

Upon admission, chest to pelvic computed tomography, 12-lead electrocardiogram, and blood tests revealed no significant abnormalities, but the laboratory test showed moderate hyponatremia (123 mEq/dL) without pancytopenia. The hyponatremia was corrected with normal saline intravenous. Infusion of 1000 ml and her serum sodium levels returned to normal (136 mEq/dL) by the ninth day of hospitalization. As she had back pain because of multiple muscle strains, she continued rehabilitation in the rehabilitation unit for the preparation of discharge to home. In the rehabilitation unit, her laboratory test did not show any pancytopenia.

On the 31st day of hospitalization, the patient developed a fever. A COVID-19 antigen test was performed to investigate the source of the fever, and the result was positive, confirming a COVID-19 infection. She had no symptoms other than fever and was considered to have a mild illness without any laboratory test abnormality, such as hyperferritinemia, high C-reactive protein, thrombocytopenia, d-dimer, or pancytopenia, which was managed with symptomatic treatment using intravenous acetaminophen because of her tachycardia (the heart rate of 110/minute) and the possibility of dehydration. Her fever subsided the following day, and she remained clinically stable.

However, on the 38th day of hospitalization (the fourth day after the COVID-19 diagnosis), she developed a persistent low-grade fever of around 37 °C. Despite periodic administration of acetaminophen, the fever persisted, and no apparent source of infection could be identified, with no specific symptoms and negative results from blood and urine culture. On the 44th day of hospitalization (the 10th day after COVID-19 diagnosis), blood tests revealed pancytopenia (neutrophil 900/dL, hemoglobin 9.1g/dL, and platelet 6.2 ×104/dL) (Table 1).

**Table 1:** The laboratory data of the patient

Parameter	Level	Reference
White blood cells	1.40	3.5-9.1 × 10^3^ /μL
Neutrophils	54.2	44.0-72.0
Lymphocytes	18.3	18.0-59.0
Hemoglobin	9.8	11.3-15.2 g/dL
Hematocrit	28.7	33.4-44.9% (in %)
Mean corpuscular volume	90.6	79.0-100.0 fl
Platelets	12.1	13.0-36.9 × 10^4^ /μL
Total protein	6.3	6.5-8.3 g/dL
Albumin	3.1	3.8-5.3 g/dL
Total bilirubin	0.7	0.2-1.2 mg/dL
Aspartate aminotransferase	426	8-38 IU/L
Alanine aminotransferase	302	4-43 IU/L
Lactate dehydrogenase	348	121-245 U/L
Blood urea nitrogen	8.3	8-20 mg/dL
Creatinine	0.58	0.40-1.10 mg/dL
Serum Na	130	135-150 mEq/L
Serum K	4.0	3.5-5.3 mEq/L
Serum Cl	96	98-110 mEq/L
C-reactive protein	4.38	0.30 mg/dL

**Figure 1:**
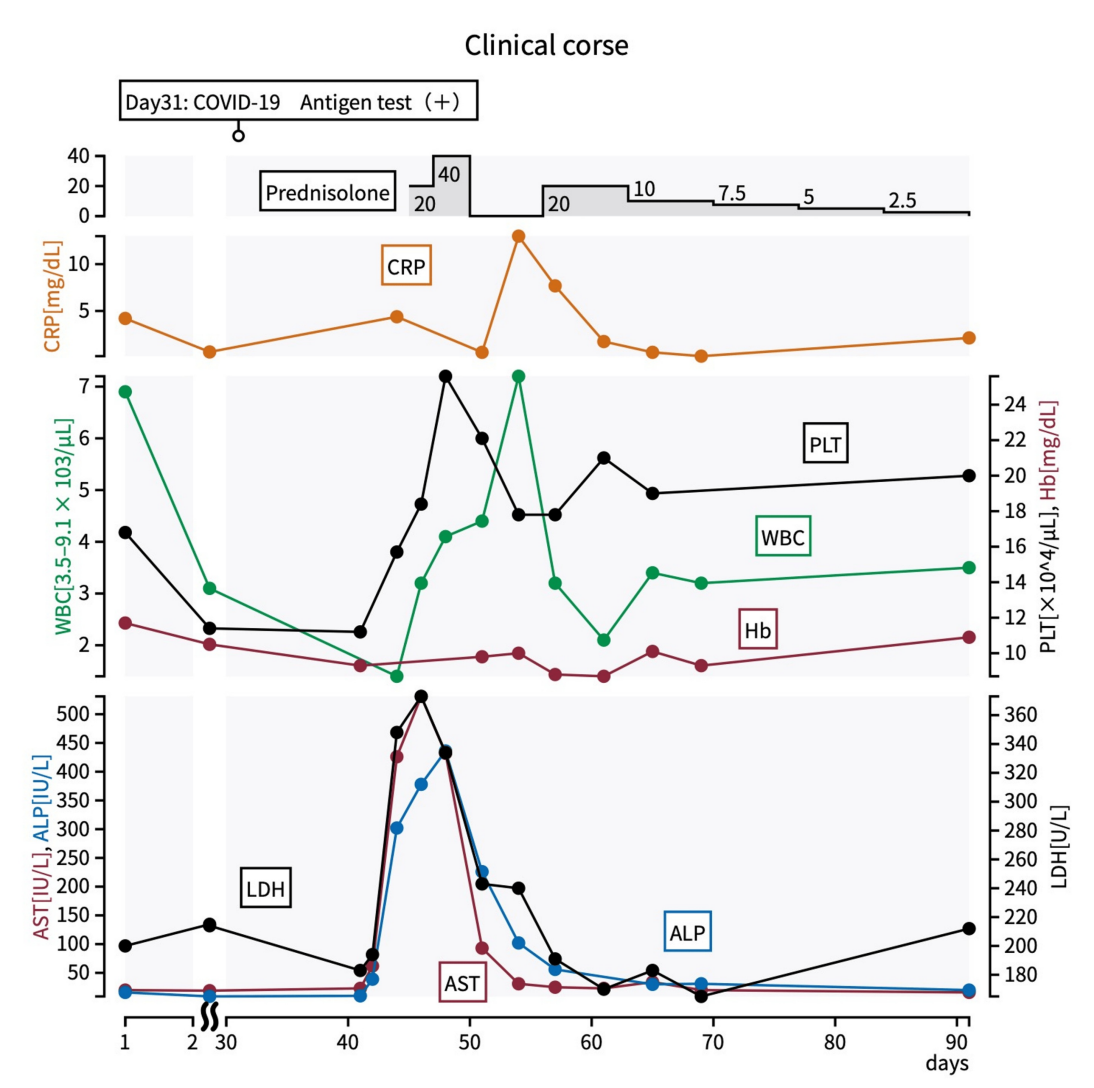
The clinical course of the case AST, aspartate aminotransferase; ALT, alanine aminotransferase; ALP, alkaline phosphatase; CRP, C-reactive protein; Hb, hemoglobin; LDH, lactate dehydrogenase; PLT, platelet; WBC, white blood cell

The next day, her temperature spiked to 38-39°C, resembling a septic fever pattern. Blood tests indicated elevated C-reactive protein and hepatobiliary enzymes (Figure 1).

**Figure 2:**
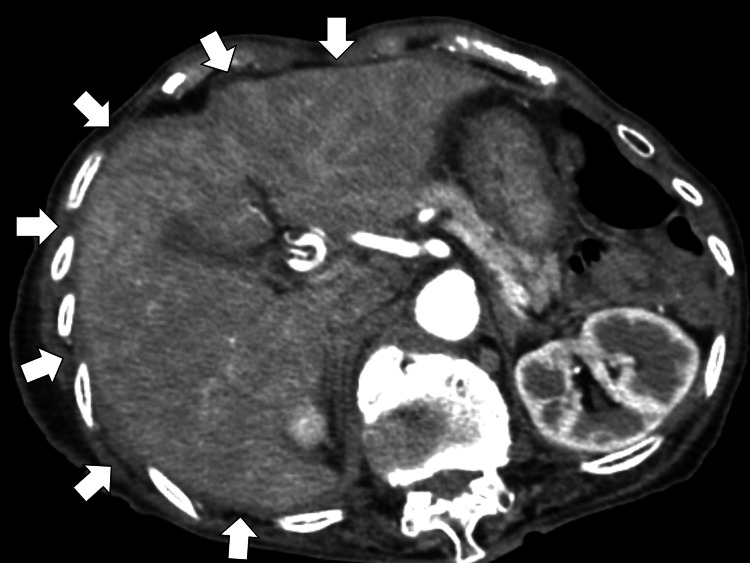
Abdominal contrast-enhanced computed tomography showing the mottled contrast effect of the liver without dilatation of the cholangial duct (white arrows)

Abdominal contrast-enhanced computed tomography was performed due to suspicion of acute cholangitis, showing the mottled contrast effect of the liver without dilatation of the cholangial duct (Figure 2). Hepatitis virus markers were negative.

Based on clinical courses and findings, the patient was diagnosed with myelosuppression and liver dysfunction secondary to multisystem inflammatory syndrome (MIS). She was started on 20 mg of prednisolone. On the 47th day of hospitalization (the 13th day after the COVID-19 diagnosis), blood tests showed further increases in hepatobiliary enzymes, prompting an increase in the prednisolone dose to 40 mg/day (Figure 1). By the 48th day of hospitalization (the 15th day after COVID-19 diagnosis), white blood cell and platelet counts began to recover, and hepatobiliary enzyme levels peaked and then decreased. Prednisolone was tapered and discontinued on the 50th day of hospitalization (17th day after COVID-19 diagnosis).

The patient’s condition remained stable, but on the 56th day of hospitalization (the 24th day after the COVID-19 diagnosis), blood tests showed recurrent pancytopenia and prednisolone 20 mg was restarted. No re-elevation of hepatobiliary enzymes was observed. Prednisolone was gradually tapered by 5 mg per week and stopped over four weeks (Figure 1). She was eventually discharged home with her previous physical and cognitive functions intact.

## Discussion

In this case, although the initial COVID-19 infection was mild, the patient experienced subsequent pancytopenia and hepatic dysfunction, accompanied by a rapid onset of fever. This case highlights the potential for MIS-A to cause severe systemic complications even after a seemingly mild COVID-19 illness. MIS-A is a rare condition characterized by non-pulmonary multisystem organ involvement, including shock, gastrointestinal symptoms, cutaneous and mucosal manifestations, and hematological abnormalities, such as thrombocytopenia, typically appearing weeks after the resolution of acute COVID-19 [5]. The pathophysiology involves an abnormal immune response triggered by systemic inflammation induced by SARS-CoV-2 infection [6].

In our patient, both liver dysfunction and pancytopenia developed after the resolution of the acute phase of COVID-19, alongside signs of systemic inflammation. Pancytopenia is a condition marked by the reduction of all three blood cell lineages - red blood cells, white blood cells, and platelets - often seen in viral infections [7]. It is known to occur in the acute phase of diseases such as Epstein-Barr, parvovirus B19, and HIV [8]. With the emergence of the COVID-19 pandemic, cases of COVID-19-associated pancytopenia have been increasingly reported [9,10]. Although the precise mechanisms remain unclear, the hyperinflammatory state resulting from the excessive production of inflammatory cytokines, such as interleukin (IL)-1, TNFα, and IL-6, is believed to contribute to myelosuppression [11]. These cytokines may inhibit the differentiation and maturation of myeloid cells, and in severe cases, cytokine storm-induced immune dysregulation can trigger hemophagocytic lymphohistiocytosis (HLH), leading to bone marrow suppression.

COVID-19 is predominantly recognized as a respiratory illness, but it can also affect multiple organs, including the liver. The mechanisms underlying COVID-19-induced liver dysfunction are multifactorial [12]. Direct viral cytotoxicity is suspected as SARS-CoV-2 proteins bind to the angiotensin-converting enzyme 2 (ACE2) receptor, expressed not only in the lungs but also in hepatocytes and bile duct cells [13]. This interaction may facilitate viral entry and directly damage these cells. Moreover, the systemic inflammatory response, often called a cytokine storm, can exacerbate liver injury [14]. Secondary factors, such as hypoxemia, shock, and adverse effects of medications used to treat COVID-19, including antivirals and antipyretics, may also contribute to hepatic dysfunction [14].

Studies from China and the United States have reported elevated liver enzymes in 14-53% of hospitalized COVID-19 patients, with elevated γ-glutamyl transpeptidase (γ-GTP), indicative of bile duct injury observed in up to 54% of cases [15]. The extent of liver damage often correlates with disease severity and may serve as a predictor of poor outcomes [16]. Although liver dysfunction in COVID-19 is generally transient and self-limiting, severe cases or those with pre-existing liver conditions, such as cirrhosis, can have a worse prognosis [15]. In our patient, the persistence of elevated liver enzymes during the post-COVID-19 phase, accompanied by general malaise, underscores the importance of monitoring liver function, especially in severe or complicated cases. Early detection and timely anti-inflammatory treatment, including corticosteroids, may improve outcomes in such scenarios.

Managing MIS-A, including corticosteroids, remains challenging, as there are no standardized treatment protocols [7]. In this case, the initial response to corticosteroids was positive, but the recurrence of symptoms after rapid tapering suggests the need for careful management. Previous studies have indicated that a gradual tapering of steroids may reduce the risk of symptom recurrence [6]. For healthcare providers, especially those in primary care, it is crucial to closely monitor patients with MIS-A for signs of recurrence during inpatient and outpatient follow-up. Adjusting steroid doses based on the patient's clinical response is essential for managing this complex condition effectively. Further case reports and studies are needed to understand better the pathophysiology, optimal treatment strategies, and long-term outcomes of MIS-A, particularly in general medicine patients with mild initial COVID-19 symptoms. This will aid in developing evidence-based guidelines for comprehensively managing such rare and severe post-COVID-19 complications in rural general medicine [17].

## Conclusions

This case highlights the potential for mild COVID-19 to progress to pancytopenia and liver dysfunction due to MIS. Cytokine storms likely drive myelosuppression and liver dysfunction in MIS. Immunosuppressive therapy with steroids has proven effective in managing these complications, but careful titration and monitoring are necessary to prevent relapse and ensure optimal outcomes. Early diagnosis, prompt treatment, and vigilant monitoring for multiorgan complications, including pancytopenia and liver dysfunction, are crucial in the management of COVID-19 patients. Appropriate management of steroid therapy may significantly influence the prognosis in such cases.
